# Association of Medicaid expansion with dental emergency department visits overall and by states’ Medicaid dental benefits provision

**DOI:** 10.1186/s12913-023-09488-3

**Published:** 2023-06-13

**Authors:** Theodoros V. Giannouchos, Julie Reynolds, Peter Damiano, Brad Wright

**Affiliations:** 1 Department of Health Services Policy & Management, Arnold School of Public Health, University of South Carolina, 915 Greene St, 29208 Columbia, SC USA; 2 Department of Preventive and Community Dentistry, College of Dentistry, University of Iowa, Iowa City, IA USA

**Keywords:** Medicaid expansion, Dental care, Emergency department, Dental benefits, Access to care

## Abstract

**Background:**

Evidence on the association of Medicaid expansion with dental emergency department (ED) utilization is limited, while even less is known on policy-related changes in dental ED visits by Medicaid programs’ dental benefits generosity. The objective of this study was to estimate the association of Medicaid expansion with changes in dental ED visits overall and by states’ benefits generosity.

**Methods:**

We used the Healthcare Cost and Utilization Project’s Fast Stats Database from 2010 to 2015 for non-elderly adults (19 to 64 years of age) across 23 States, 11 of which expanded Medicaid in January 2014 while 12 did not. Difference-in-differences regression models were used to estimate changes in dental-related ED visits overall and further stratified by states’ dental benefit coverage in Medicaid between expansion and non-expansion States.

**Results:**

After 2014, dental ED visits declined by 10.9 [95% confidence intervals (CI): -18.5 to -3.4] visits per 100,000 population quarterly in states that expanded Medicaid compared to non-expansion states. However, the overall decline was concentrated in Medicaid expansion states with dental benefits. In particular, among expansion states, dental ED visits per 100,000 population declined by 11.4 visits (95% CI: -17.9 to -4.9) quarterly in states with dental benefits in Medicaid compared to states with emergency-only or no dental benefits. Significant differences between non-expansion states by Medicaid’s dental benefits generosity were not observed [6.3 visits (95% CI: -22.3 to 34.9)].

**Conclusions:**

Our findings suggest the need to strengthen public health insurance programs with more generous dental benefits to curtail costly dental ED visits.

**Supplementary Information:**

The online version contains supplementary material available at 10.1186/s12913-023-09488-3.

## Introduction

In the United States, over 2 million emergency department (ED) visits for dental-related conditions occurred in 2018, accounting for 2.5% of all ED visits and totaling more than $2 billion nationally [1, 2]. Individuals with limited or no access to regular dental care often use hospital EDs to seek care for non-traumatic oral health problems, which are better cared for in a dental office setting and, if left untreated, can result in adverse and even life-threatening outcomes [1, 3, 4]. Dental-related ED visits among adults are more common among those who are uninsured or enrolled in Medicaid, particularly in states with limited Medicaid dental benefits for adults, and those who live in areas with a lower supply of dental providers [2, 5–8]. Most often, the dental care provided in the ED is non-definitive, meaning the ED is not able to treat the cause of the problem. Rather, the ED physicians prescribe pain medications and/or antibiotics, with the tooth extraction or root canal still needing to be provided in a dental office following the ED visit [9].

Expanding Medicaid eligibility to non-elderly adults with incomes up to 138% of the federal poverty level through the Patient Protection and Affordable Care Act of 2010 (ACA) provided millions of previously uninsured Americans with health insurance coverage through Medicaid [10–15]. Since then, Medicaid expansion has been associated with a healthcare system-wide increase in the use of preventive services, access to primary care providers, affordability, and quality of care [10, 12, 15, 16–18].

Gains in Medicaid enrollment through the expansion of Medicaid have also been associated with improved access to routine and preventive dental care and improved oral health outcomes [19–24]. However, since the provision of adult dental benefits in Medicaid is optional, many states offer no or only emergency dental benefits to Medicaid enrollees, while others offer more comprehensive and generous dental benefits. As of 2021, three states provided no adult dental benefits, nine provided coverage for dental care only in the case of an emergency (e.g., pain or infection), and 37 provided more than emergency-only coverage [25]. There is evidence indicating that access to dental care has disproportionately improved in states that expanded Medicaid and offered more generous dental benefits in Medicaid compared to those that did not [19, 20, 24, 26, 27]. These findings suggest that Medicaid expansion’s impact on access to dental care is contingent on the provision of adult dental benefits.

Despite growing evidence of the impacts of Medicaid expansion and the provision of adult dental benefits on access to regular dental care, only a few studies have assessed the association of Medicaid expansion with dental-related ED visits.[5, 28–31] Studies focusing on single-state data have yielded mixed results, with some finding that dental ED visits increased after Medicaid expansion, while others observed declines, particularly in ED visits for non-traumatic dental conditions [5, 28–30]. Recent work found that dental ED visits decreased by 14.1% in Medicaid expansion states with more generous Medicaid dental benefits for adults, while ED visits rose in both expansion states without dental benefits and non-expansion states [31]. However, this study was limited to only two years of hospital data from eight states (2012: pre-expansion year; 2014: post-expansion year) [31]. Hence, there remains a need for analyses to examine if Medicaid expansion was associated with changes in dental ED visits overall, stratified by states’ variation in Medicaid adult dental benefit generosity using data across multiple states and years.

To address this gap in the literature, the purpose of this study was to estimate the association between Medicaid expansion and dental ED visits and changes in ED visits’ payer mix by comparing Medicaid expansion and non-expansion states. We also examined the extent to which this relationship varied as a function of differing levels of dental benefit generosity across state Medicaid programs. We hypothesized that dental ED visits would decline only in states that both expanded Medicaid and offered more generous dental benefits in their Medicaid programs in post-expansion period, since expanding eligibility for health insurance coverage does not guarantee access to dental care. Using six years of data from 23 states, our study represents the most comprehensive analysis of the relationship between Medicaid expansion and dental ED visits to date. Our findings provide insight to both state and federal policymakers and other stakeholders as they contemplate future policy initiatives designed to increase access to dental care, improve oral health outcomes, and contain healthcare costs.

## Methods

### Study design and data source

We used retrospective, longitudinal data from the Agency for Healthcare Research and Quality’s (AHRQ) Healthcare Cost and Utilization Project (HCUP) Fast Stats database, which is a publicly available database that provides aggregate quarterly counts of ED visits for dental conditions rounded to the nearest 50 ED visits for participating states [32]. These ED visits include both outpatient (treat and release) visits and ED visits that subsequently resulted in hospital admissions. We included dental ED visits for non-elderly adults (19 to 64) with the expected primary payer listed as Medicaid, private plans, or self-pay/no charge (referred to as uninsured hereafter) since these individuals were most likely to be affected by Medicaid expansion [31, 33, 34]. Dental ED visits were defined by AHRQ using the International Classification of Diseases Ninth Revision, Clinical Modification (ICD-9-CM) diagnosis codes 520.0 to 523.9 based on the principal or first-listed diagnosis in each encounter [32].

We analyzed dental ED visits across 23 states, 11 of which expanded Medicaid in January 2014 while 12 did not [35]. We used 15 quarters before (2010, 2011, 2012, 2013 up to quarter three) and 7 corresponding quarters after Medicaid expansion (2014 & 2015 quarter three) in our analyses, a period over which all 23 states submitted data on dental ED visits. Like previous work, we dropped the fourth quarter of 2013 from the analysis, because many newly Medicaid-eligible individuals enrolled in Medicaid after discovering eligibility during the Health Insurance Marketplace open enrollment period in late 2013 [33]. We further restricted our analysis up to the third quarter of 2015 due to changes in the International Classification of Diseases (ICD), Clinical Modification coding system from the 9th to the 10th version in the fourth quarter of 2015 which resulted in documented increases in dental ED visits by more than 50% on average after the coding system change [32]. However, these increases reflect definitional rather than ED utilization changes [32]. Additionally, the 23 states in our analyses also had consistent Medicaid dental benefit coverage policies for adults during this period. We obtained and combined information on the coverage categories of adult state-level Medicaid dental benefits from publicly available resources [36–38]. Consistent with previous work, we compared states that provided more than emergency Medicaid dental benefits for adults (i.e., limited or extensive dental coverage, 14 states) with states that provided emergency-only or no dental benefits (9 states) [31].

### Outcomes of interest

Our outcomes of interest were dental ED visits per 100,000 population of non-elderly adults who were covered through Medicaid or private health insurance plans or lacked coverage throughout the study years. We also analyzed changes in payer mix (Medicaid, private plans, and uninsured) calculated as the share of payer-specific adult dental ED visits of the sum of ED visits by these three payers.

### Covariates

We adjusted for publicly available, time-varying, population, and state-level variables in our analyses that are associated with overall ED use and may also be associated with dental ED visits (percentage female, percentage non-Hispanic Black, percentage Hispanic, percentage of population aged 35 to 64 years, percentage of population within 0–100% and 100–200% of the Federal Poverty Level, unemployment rate, percentage of population uninsured, and the number of hospitals and dentists per 100,000 population) [31, 39–40].

### Statistical analysis

We initially conducted a descriptive analysis for all 23 states stratified by Medicaid expansion status. We then conducted two-way fixed effects difference-in-differences regression analyses to estimate the association between Medicaid expansion and dental ED visits. Similarly, we examined the relationship between Medicaid expansion and changes in the dental ED visits payer mix. This enabled us to compare pre- versus post-Medicaid expansion outcomes in states that implemented the expansion (‘treatment’ group) to states that did not (‘control’ group). We then stratified expansion and non-expansion states based on their Medicaid programs’ adult dental benefits generosity and conducted similar regressions. We used multivariable ordinary least squares regressions for panel data to implement the difference-in-differences analyses. Our adjusted regression analyses included all covariates described above, as well as year-quarter and state-fixed effects. We used robust standard errors clustered at the state level. The adjusted difference-in-differences models were specified as:

$$ {Y}_{it}= {\beta }_{0}+{\beta }_{1}{Expansion}_{it}+\lambda {X}_{it}+{\delta }_{i}+{\gamma }_{t}+{\epsilon }_{it}$$

where $$ {Y}_{it}$$ are the total dental ED visits per 100,000 population at the state-quarter level (state *i* in quarter *t*) and $$ {Expansion}_{it}$$ is a binary variable equal to 1 if state *i* has expanded Medicaid in that quarter and 0 otherwise. Hence $$ {\beta }_{1}$$ is the difference-in-differences coefficient of interest that indicates the pre-and post-policy implementation difference between states that expanded Medicaid compared to those that did not. The vector $$ {X}_{it}$$ includes the observed time-varying covariates, while $$ {\delta }_{i}$$ are state fixed effects and $$ {\gamma }_{t}$$ are year-quarter (time) fixed effects to control for unobserved state-specific time-invariant factors and for time-specific unobserved factors respectively.

One critical assumption of the difference-in-differences model that enables unbiased assessment of the causal impact of a policy is that both the treatment and control groups exhibited parallel trends in the pre-policy implementation period. Although this assumption cannot be tested directly, we examined trends in the pre-Medicaid expansion period by conducting event studies and interacting the dichotomous expansion variable (0 = no expansion; 1 = expansion) with the number of quarters used in our study in adjusted regressions. Statistically insignificant coefficients between expansion and non-expansion states before the Medicaid expansion period would suggest no apparent differences between the two groups and thus that the parallel trend assumption holds. The event studies also enabled us to examine the treatment effects of the Medicaid expansion over time after 2014. Since all expansion states in our sample expanded Medicaid in January 2014, variation in treatment timing did not bias our regression estimates [42, 43]. We managed the data and conducted all statistical analyses using Stata version 17.0 (StataCorp, College Station, TX). The study included publicly available, aggregate, state-level data and was determined to be not human subjects research by the University of South Carolina Institutional Review Board.

## Results

### Dental ED visits and states’ characteristics by Medicaid expansion status

Our study included 2.0 million dental ED visits across 23 states. Table 1 presents descriptive information on dental ED visits and population and state-level characteristics for all states stratified by Medicaid expansion status. On average, quarterly dental ED visits were lower in Medicaid expansion states versus non-expansion states by 1412.9 ED visits overall (3213.2 versus 4626.1, *P* < 0.001) and 25.0 ED visits per 100,000 population (104.5 versus 129.5, *P* < 0.001) throughout the study period. The share of Medicaid-paid dental ED visits was disproportionately higher in expansion states versus non-expansion states (43.1% versus 31.2%, *P* < 0.001), while the opposite was observed for the share of the uninsured (36.2% versus 48.6%, *P* < 0.001). No difference was observed in the share of dental ED visits paid by private plans across expansion and non-expansion states (20.7% versus 20.2%, *P* = 0.572).

**Table 1:** Descriptive statistics for all states and stratified by Medicaid expansion status among non-elderly adults with Medicaid, private, or no health insurance coverage

	Non-expansion States (N = 12)	Expansion States (N = 11)	*P*
Total number of dental ED visits	1,221,300	777,600	
Total quarterly visits, state average	4626.1 (3976.7)	3213.2 (2588.2)	< 0.001
Total quarterly visits per 100,000, state average	129.5 (62.1)	104.5 (36.6)	< 0.001
Share of dental ED visits by payer source (%)			
Medicaid	31.2 (11.5)	43.1 (15.4)	< 0.001
Uninsured	48.6 (12.6)	36.2 (15.8)	< 0.001
Private insurance	20.2 (11.6)	20.7 (7.0)	0.572
Population / State-level characteristics			
Female (%)	48.9	49.1	0.040
Race/Ethnicity (%)			
Non-Hispanic whites	74.7	70.8	0.001
Non-Hispanic blacks	11.8	8.7	< 0.001
Hispanic	8.3	12.9	< 0.001
Age group 35 to 64 years of age	39.5	40.8	< 0.001
Uninsured share of non-elderly population (%)	19.7	15.0	< 0.001
Unemployment rate	6.8	7.3	0.012
Population shares by poverty level (%)			
0–100% pf FPL	15.3	13.6	< 0.001
100–200% of FPL	19.9	17.1	< 0.001
Number of hospitals per 100,000 population	4.5	2.8	< 0.001
Number of dentists per 100,000 population	52.2	62.5	< 0.001
Number of states that offer adult dental benefits in Medicaid	5	9	
State population of non-elderly adults with Medicaid, private, or no health insurance (millions), average	3.4	3.5	0.718

**Notes** Authors’ analysis of the HCUP Fast Stats Databases for 2010, 2011, 2012, 2013 up to quarter 3, 2014, and 2015 up to quarter 3 of individuals ages 19–64 who were covered by Medicaid, private plans, or were uninsured. The table reflects data for 23 states; 11 states expanded Medicaid in January 2014: Arizona, Iowa, Kentucky, Maryland, Massachusetts, Minnesota, Nevada, New Jersey, New York, Rhode Island, Vermont; 12 states did not expand Medicaid in January 2014: Florida, Georgia, Kansas, Maine, Missouri, Nebraska, North Carolina, South Carolina, South Dakota, Tennessee, Utah, Wisconsin. Population / State characteristics were obtained from publicly available resources. States with limited or extensive adult dental benefits in Medicaid were considered as offering benefits

The population and state-level characteristics between Medicaid expansion and non-expansion states also differed, with Medicaid expansion states having higher proportions of Hispanic population, unemployment rate, and number of dentists per 100,000 population. The opposite was observed for non-Hispanic Black and White populations, the uninsured, low-income individuals, and hospital supply. The average state population of non-elderly adults with Medicaid, private, or no health insurance coverage was similar between expansion and non-expansion states (3.5 million versus 3.4 million, *P* = 0.718). Overall, 9 out of 12 Medicaid expansion states offered generous (limited or extensive) adult dental benefits in their Medicaid programs compared to 5 out of 11 non-expansion states.

### Difference-in-differences analyses

Table 2 shows both unadjusted and regression-adjusted difference-in-differences results. Overall, the total number of quarterly dental ED visits per 100,000 population increased by 3.9% from the pre to the post- Medicaid expansion period in non-expansion states (net difference = 5.0; from 127.9 to 132.9), but decreased by 2.8% in Medicaid expansion states (net difference = − 2.9; from 105.4 to 102.5 visits). This resulted in a significant decline of 10.9 population-adjusted dental ED visits (95% CI = − 18.5 to − 3.4) per quarter after 2014 in states that expanded Medicaid compared to states that did not relative to the pre-expansion period, according to the adjusted regression-based estimates. In expansion states, the share of Medicaid-paid adult dental ED visits increased by 18.8 percentage points after 2014, while it remained relatively unchanged in non-expansion states, resulting in a significant 15.9 percentage point increase in Medicaid-paid adult ED visits (95% CI = 11.3 to 20.5) associated with the Medicaid expansion. In contrast, the Medicaid expansion was associated with 10.9 (95% CI = − 16.6 to − 5.2) and 5.0 (95% CI = − 8.6 to − 1.4) percentage point declines in the shares of dental ED visits by the uninsured and the privately insured respectively.

**Table 2:** Difference-in-differences (DiD) regression analyses: changes in quarterly dental ED visits per 100,000 population overall and by Medicaid programs’ dental benefit generosity, and in payer-mix

Dental ED visits, per quarter	Pre-expansion period		Post-expansion period	Difference	Unadjusted DiD	Adjusted DiD (95% CI)
**Total per 100,000**					-7.9	-10.9 (-18.5 − -3.4)
Non-expansion States	127.9		132.9	5.0		
Expansion States	105.4		102.5	-2.9		
**Medicaid share**					19.2	15.9 (11.3–20.5)
Non-expansion States	31.3		30.9	-0.4		
Expansion States	37.1		55.9	18.8		
**Uninsured share**					-15.5	-10.9 (-16.6 − -5.2)
Non-expansion States	49.6		46.2	-3.4		
Expansion States	42.2		23.3	-18.9		
**Private insurance share**					-3.6	-5.0 (-8.6 – -1.4)
Non-expansion States	19.0		22.9	3.9		
Expansion States	20.6		20.9	0.3		
**Medicaid dental benefits generosity**						
Non-expansion States					-3.2	6.3 (-22.3–34.9)
Emergency-only or none	155.5		161.8	6.3		
More than emergency	89.3		92.4	3.1		
Expansion States					-7.2	-11.4 (-17.9 – -4.9)
Emergency-only or none	78.0		81.0	3.0		
More than emergency	111.5		107.3	-4.2		

**Notes** Authors’ analysis of the HCUP Fast Stats Databases for 2010, 2011, 2012, 2013 up to quarter 3, 2014, and 2015 up to quarter 3 of individuals ages 19–64 who were covered by Medicaid, private plans, or were uninsured. The analysis contained 22 quarters for each of the 23 states. Of those, 15 state-year-quarters correspond to the pre-Medicaid expansion years (2010 quarter 1 to 2013 quarter 3). 11 states expanded Medicaid in January 2014: Arizona, Iowa, Kentucky, Maryland, Massachusetts, Minnesota, Nevada, New Jersey, New York, Rhode Island, Vermont; 12 states did not expand Medicaid in January 2014: Florida, Georgia, Kansas, Maine, Missouri, Nebraska, North Carolina, South Carolina, South Dakota, Tennessee, Utah, Wisconsin. 14 states offered more than emergency dental benefits in their Medicaid programs: Iowa, Kansas, Kentucky, Maryland, Massachusetts, Minnesota, Nebraska, New Jersey, New York, North Carolina, Rhode Island, South Dakota, Vermont, Wisconsin; 9 states offered emergency or no dental benefits in their Medicaid programs: Arizona, Florida, Georgia, Maine, Missouri, Nevada, South Carolina, Tennessee, Utah. Results show differences-in-differences estimates for expansion states versus non-expansion states and by Medicaid programs’ dental benefits generosity. Adjusted analyses controlled for percent female, percent non-Hispanic black, percent Hispanic, percent of population aged 35 to 64 years, percent of population with 0–100% of FPL, percent of population with 100–200% of FPL, unemployment rate, number of hospitals per 100,000 population, number of dentists per 100,000 population, and percent of population with no health insurance coverage

**Fig. 1:**
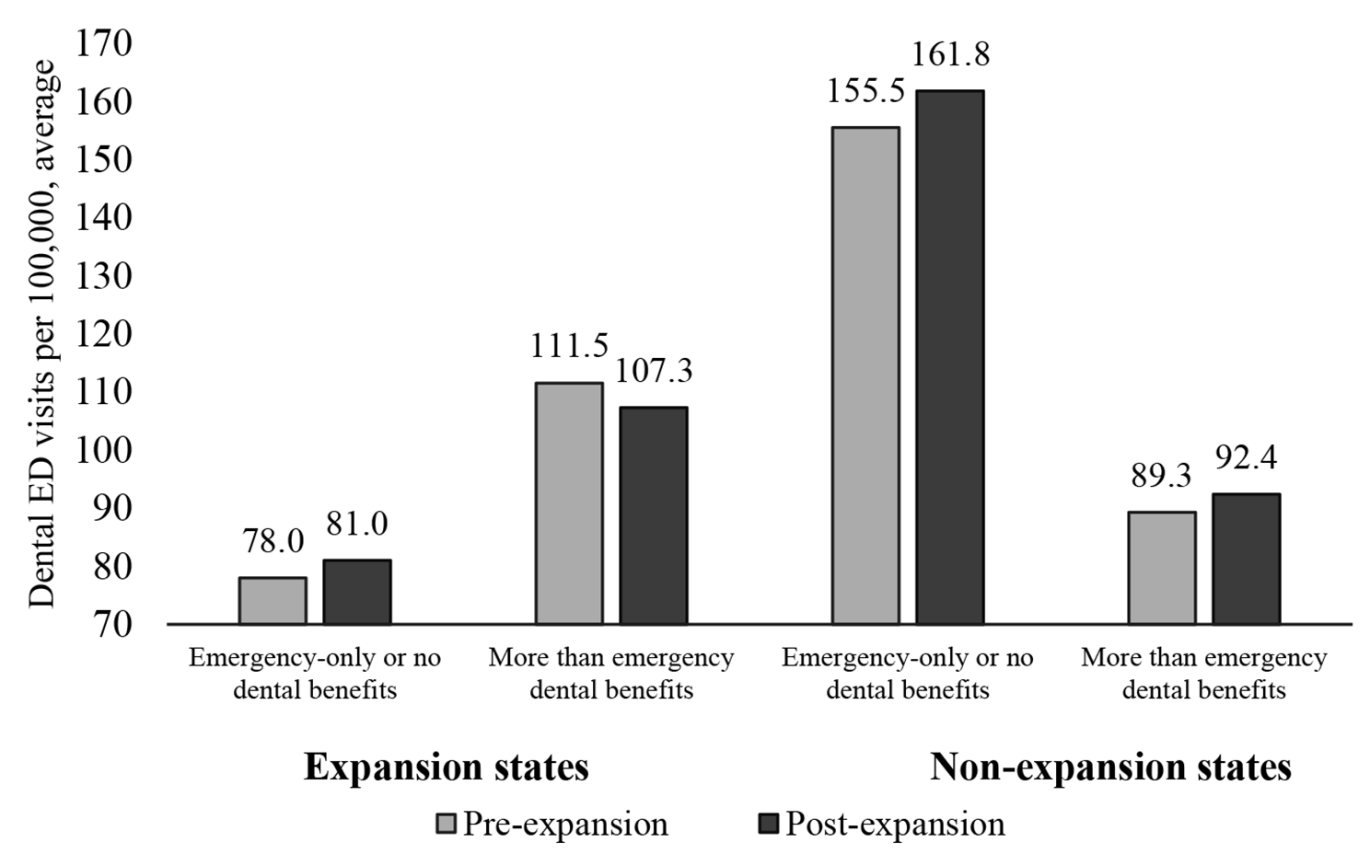
Emergency department dental visits per 100,000 population by states’ Medicaid expansion and adult dental benefit generosity status before and after 2014 **Notes** Authors’ analysis of the HCUP Fast Stats Databases for 2010, 2011, 2012, 2013 up to quarter 3, 2014, and 2015 up to quarter 3 of individuals ages 19–64 who were covered by Medicaid, private plans, or were uninsured. The analysis contained 22 quarters for each of the 23 states. Of those, 15 state-year-quarters correspond to the pre-Medicaid expansion years (2010 quarter 1 to 2013 quarter 3). 11 states expanded Medicaid in January 2014: Arizona, Iowa, Kentucky, Maryland, Massachusetts, Minnesota, Nevada, New Jersey, New York, Rhode Island, Vermont; 12 states did not expand Medicaid in January 2014: Florida, Georgia, Kansas, Maine, Missouri, Nebraska, North Carolina, South Carolina, South Dakota, Tennessee, Utah, Wisconsin. 14 states offered more than emergency dental benefits in their Medicaid programs: Iowa, Kansas, Kentucky, Maryland, Massachusetts, Minnesota, Nebraska, New Jersey, New York, North Carolina, Rhode Island, South Dakota, Vermont, Wisconsin; 9 states offered emergency or no dental benefits in their Medicaid programs: Arizona, Florida, Georgia, Maine, Missouri, Nevada, South Carolina, Tennessee, Utah. Y-axis shows average dental ED visits per 100,000 population per quarter

The stratified analyses by Medicaid dental benefits generosity for adults in expansion states indicated that dental ED visits declined by 4.2 quarterly visits per 100,000 population in states that offered more than emergency benefits in the post-expansion period, while dental ED visits per 100,000 population increased by 3.0 visits per quarter after 2014 in expansion states which offered emergency-only or no dental benefits, relative to the pre-expansion period (Table 2; Fig. 1). This resulted in a significant regression-adjusted decline of 11.4 (95% CI=-17.9 to -4.9, p < 0.001) quarterly dental ED visits per 100,000 population further associated with dental benefits generosity in expansion states after 2014 (Table 2). We did not observe any differences between non-expansion states in the post-expansion period based on Medicaid programs’ dental benefits provision (6.3, 95% CI=-22.3 to 34.9, *P* = 0.666).

### Event studies’ regression estimates

The treatment effect coefficients of the adjusted event studies’ regressions revealed no significant differences between expansion and non-expansion states in the pre-Medicaid expansion period across all outcomes, suggesting that the parallel trends assumption was satisfied (Supplementary Material Fig. 1). However, after Medicaid expansion, we found significant declines in total dental ED visits, in the uninsured and privately insured shares of dental ED visits in expansion versus non-expansion states, while Medicaid shares increased, consistent with our main analyses (Supplementary Material Fig. 1).

## Discussion

In our analysis of 2.0 million ED visits among non-elderly adults with Medicaid, private health insurance, or who were uninsured across 23 states, we found that the ACA’s Medicaid eligibility expansion was associated with a quarterly decline of 10.9 dental ED visits per 100,000 population in states that expanded Medicaid in 2014 compared to states that did not. We also documented changes in payer mix for dental ED visits, with policy-related increases in the Medicaid share, and corresponding declines in the shares of private health insurance and the uninsured. Among Medicaid expansion states, the provision of more generous Medicaid dental benefits for adults was further associated with 11.4 fewer quarterly dental ED visits per 100,000 population compared to states that offered emergency-only or no dental benefits. We did not observe any policy-related differences in quarterly dental ED visits based on dental benefit generosity in non-expansion states.

Our results are consistent with previous work that documented decreases in both overall and dental-related ED visits and similar changes in payer-mix following the expansion of Medicaid in states that implemented the policy compared to those that did not [15, 31, 33, 34, 44–47]. We further found that dental-related ED visits declined in Medicaid expansion states with more generous adult dental benefits compared to those with emergency-only or no dental benefits. These results are in-line with previous studies that documented increases in the use of regular dental care and preventive services in states with generous adult dental benefits, while a study in Minnesota, a state with more than emergency dental benefits in Medicaid, found declines in ED visits for non-traumatic dental conditions following the expansion of Medicaid [8, 19, 20, 22, 23]. Another study using data from ED visits in California observed increases in dental-related ED visits following the elimination of Medicaid adult dental benefits [5]. These results suggest that when dental coverage is restricted or eliminated, patients with Medicaid may either turn to the ED —rather than a dental office— for their care, or they may simply delay care until it becomes emergent.

Our findings extend the current literature by providing evidence that the decline in ED visits for dental conditions was mostly concentrated in states that expanded Medicaid and offered more generous adult dental benefits in their Medicaid programs. This finding suggests that health insurance coverage alone might not be sufficient to increase access to dental care among new Medicaid enrollees and to contain costly and potentially preventable dental-related ED visits. Our findings further suggest that policymakers should work to ensure that states with generous Medicaid dental benefits for adults make maintaining those benefits a priority, while other states should strongly consider the advantages of enhancing less comprehensive Medicaid-covered dental benefits and complement health insurance coverage expansions [5, 26, 30]. The validity of our findings was supported by the lack of policy-related changes in ED visits in non-expansion states independent of their Medicaid dental benefits coverage.

Dental-related ED visits by Medicaid enrollees and the uninsured made up more than 60% of all ED visits by non-elderly adults. Expanding health insurance coverage and including dental benefits in public health insurance programs does not guarantee access to oral care and is unlikely to reverse dental-related ED visits without addressing prevailing barriers to dental providers. Prevailing socioeconomic needs and infrastructure barriers have been commonly associated with ED utilization for dental conditions, particularly among lower-income populations [2, 6, 7, 48]. For example, a recent study found that about 11% of Medicaid-insured adults reported unmet dental needs due to transportation barriers, which were associated with decreased use of dental services [48]. Additionally, there is further evidence that increased uptake of dental services following Medicaid expansion has been mostly concentrated in expansion states with generous Medicaid dental benefits and a greater supply of dentists [20].

Furthermore, there are large state-wide variations in the number of dentists currently enrolled as Medicaid providers and in the volume of Medicaid patients seen by participating providers [49]. According to a research brief from the American Dental Association’s Health Policy Institute, the share of dentists who were enrolled as Medicaid providers ranged from 13% in New Hampshire to 89% in Iowa in 2017 [49]. Moreover, among dentists enrolled as Medicaid providers in the same year, more than half of these providers treated zero to nine Medicaid patients, while only about 20% of dentists saw more than 100 Medicaid patients overall, ranging from 3% in Maine up to 34% in Vermont [49]. Non-Medicaid participating dentists have reported low fees, denial of payments, broken appointments by patients, complicated paperwork, and social stigma from other dentists as their main reasons for not participating in Medicaid programs [50–53]. Hence, higher Medicaid payment levels and reduced administrative complexity are also critical to enhance the supply of dentists in Medicaid, beyond addressing social and health insurance coverage factors, ranging from expansion of public plans to inclusion of more generous dental benefits, to reverse factors that exacerbate access to oral health and predispose costly and non-definitive dental care in the EDs. Future work using more detailed patient-level dental ED visit data beyond 2015 is needed, as is work examining how dentists’ participation in Medicaid and the volume of patients they see might be associated with dental ED visits.

Our study has several limitations. First, participation in the HCUP Fast Stats tool is voluntary, and not all states submit ED data to Fast Stats. Because we used data for 23 states, our findings might not be generalizable nationally and reflect the association of Medicaid expansion with dental ED visits in all states. However, the states used in our analyses are sociodemographically diverse and constitute almost half of the US population across all four regions of the country. Second, we were not able to use data beyond the third quarter of 2015 due to the changes in the ICD coding system from the 9th to the 10th version in the fourth quarter of 2015 which have resulted in documented increases in dental ED visits due to definitional reasons [32]. Third, we were unable to explore potential variations in dental ED visits by the number of dentists that are enrolled as Medicaid providers and the volume of Medicaid patients that enrolled providers see each year in combination with dental benefits generosity, due to the absence of such information over the study period. Fourth, we were not able to stratify analyses based on non-traumatic dental conditions (NTDC) or traumatic dental visits because all dental-related visits were provided in aggregate. NTDC ED visits are an indicator of poor access to care because they are treatable in a dental office and would be expected to be influenced by Medicaid policies to a greater degree than traumatic dental visits. Finally, we used a retrospective, quasi-experimental design which does not enable causal inferences, despite the use of a concurrent control group and the satisfaction of the parallel trends assumption, and the wide use of such methods for health policy evaluation.

## Conclusion

Our findings suggest that Medicaid expansion was associated with significant reductions in adult dental ED visits in expansion states with more generous Medicaid dental benefits for adults. Nevertheless, adult Medicaid beneficiaries and the uninsured accounted for disproportionately higher shares of dental ED visits relative to their population prevalence. Policymakers should consider the need to promote dentists’ Medicaid participation, to complement health insurance coverage expansions with more comprehensive dental benefits in Medicaid, and to address access to dental care for the uninsured to improve oral health outcomes and reduce the volume of costly and potentially preventable dental ED visits.

## Electronic supplementary material

Below is the link to the electronic supplementary material.

Supplementary Material 1

## Data Availability

The datasets generated and /or analyzed during the current study are publicly available and can be accessed at https://datatools.ahrq.gov/hcup-fast-stats#downloads.

## List of abbreviations

ACA: Affordable Care Act
AHRQ: Agency for Healthcare Research and Quality
CI: confidence interval
DiD: Difference-in-differences
ED: emergency department
HCUP: Healthcare Cost and Utilization Project
ICD: International Classification of Diseases
NTDC: non-traumatic dental conditions
