# Correction: Association of Medicaid expansion with dental emergency department visits overall and by states’ Medicaid dental benefits provision

**DOI:** 10.1186/s12913-023-09709-9

**Published:** 2023-06-27

**Authors:** Theodoros V. Giannouchos, Julie Reynolds, Peter Damiano, Brad Wright

**Affiliations:** 1 Department of Health Services Policy & Management, Arnold School of Public Health, University of South Carolina, 915 Greene St, Columbia, SC 29208 USA; 2 Department of Preventive and Community Dentistry, College of Dentistry, University of Iowa, Iowa City, IA USA

**Correction: BMC Health Serv Res 23, 625 (2023)**

**https://doi.org/10.1186/s12913-023-09488-3**

Following publication of the original article [1], the authors found a typesetting error in Table 1. The incomplete table was erroneously published in the PDF version of the article. The complete Table 1 is given in this correction article and the original article [1] has been corrected.

**Table 1:** Descriptive statistics for all states and stratified by Medicaid expansion status among non-elderly adults with Medicaid, private, or no health insurance coverage

	**Non-expansion States (*N* = 12)**	**Expansion States (*N* = 11)**	***P***
Total number of dental ED visits	1,221,300	777,600	
Total quarterly visits, state average	4626.1 (3976.7)	3213.2 (2588.2)	< 0.001
Total quarterly visits per 100,000, state average	129.5 (62.1)	104.5 (36.6)	< 0.001
Share of dental ED visits by payer source (%)			
Medicaid	31.2 (11.5)	43.1 (15.4)	< 0.001
Uninsured	48.6 (12.6)	36.2 (15.8)	< 0.001
Private insurance	20.2 (11.6)	20.7 (7.0)	0.572
Population / State-level characteristics			
Female (%)	48.9	49.1	0.040
Race/Ethnicity (%)			
Non-Hispanic whites	74.7	70.8	0.001
Non-Hispanic blacks	11.8	8.7	< 0.001
Hispanic	8.3	12.9	< 0.001
Age group 35 to 64 years of age	39.5	40.8	< 0.001
Uninsured share of non-elderly population (%)	19.7	15.0	< 0.001
Unemployment rate	6.8	7.3	0.012
Population shares by poverty level (%)			
0–100% pf FPL	15.3	13.6	< 0.001
100–200% of FPL	19.9	17.1	< 0.001
Number of hospitals per 100,000 population	4.5	2.8	< 0.001
Number of dentists per 100,000 population	52.2	62.5	< 0.001
Number of states that offer adult dental benefits in Medicaid	5	9	
State population of non-elderly adults with Medicaid, private, or no health insurance (millions), average	3.4	3.5	0.718

Notes: Authors’ analysis of the HCUP Fast Stats Databases for 2010, 2011, 2012, 2013 up to quarter 3, 2014, and 2015 up to quarter 3 of individuals ages 19–64 who were covered by Medicaid, private plans, or were uninsured. The table reflects data for 23 states; 11 states expanded Medicaid in January 2014: Arizona, Iowa, Kentucky, Maryland, Massachusetts, Minnesota, Nevada, New Jersey, New York, Rhode Island, Vermont; 12 states did not expand Medicaid in January 2014: Florida, Georgia, Kansas, Maine, Missouri, Nebraska, North Carolina, South Carolina, South Dakota, Tennessee, Utah, Wisconsin. Population / State characteristics were obtained from publicly available resources. States with limited or extensive adult dental benefits in Medicaid were considered as offering benefits
