# Supplementary material for: Association of Medicaid expansion with dental emergency department visits overall and by states’ Medicaid dental benefits provision
Source: BMC Health Serv Res. 2023 Jun 13;23:625. doi: 10.1186/s12913-023-09488-3 (PMC10262360; doi:10.1186/s12913-023-09488-3)
Supplement: Supplementary file 1 — Supplementary Material 1 [file 12913_2023_9488_MOESM1_ESM.docx]

**Appendix Figure 1:** Event study regressions’ treatment effect estimates for each outcome variable between Medicaid-expansion and non-expansion states overtime

**Notes** Authors’ analysis of the HCUP Fast Stats Databases for 2010, 2011, 2012, 2013 up to quarter 3, 2014, and 2015 up to quarter 3 of individuals ages 19-64 who were covered by Medicaid, private plans, or were uninsured. The analysis contained 22 quarters for each of the 23 states. Of those, 15 state-year-quarters correspond to the pre-Medicaid expansion years (2010 quarter 1 to 2013 quarter 3). 11 states expanded Medicaid in January 2014: Arizona, Iowa, Kentucky, Maryland, Massachusetts, Minnesota, Nevada, New Jersey, New York, Rhode Island, Vermont; 12 states did not expand Medicaid in January 2014: Florida, Georgia, Kansas, Maine, Missouri, Nebraska, North Carolina, South Carolina, South Dakota, Tennessee, Utah, Wisconsin. 14 states offered more than emergency dental benefits in their Medicaid programs: Iowa, Kansas, Kentucky, Maryland, Massachusetts, Minnesota, Nebraska, New Jersey, New York, North Carolina, Rhode Island, South Dakota, Vermont, Wisconsin; 9 states offered emergency or no dental benefits in their Medicaid programs: Arizona, Florida, Georgia, Maine, Missouri, Nevada, South Carolina, Tennessee, Utah. Results show treatment effects for each quarter between expansion states and non-expansion states before and after the expansion of Medicaid and by dental benefits generosity within Medicaid expansion states from the baseline quarter (first quarter of 2011). Y-axis indicates absolute changes. X-axis indicates quarters, with values <0 referring to pre-expansion quarters; 0 referring to the first quarter of 2014; values>0 referring to quarters after the first quarter of 2014. The dotted lines indicate ±1 standard deviation confidence intervals. Adjusted analyses controlled for percent female, percent non-Hispanic black, percent Hispanic, percent of population aged 35 to 64 years, percent of population with 0% to 100% of FPL, percent of population with 100% to 200% of FPL, number of hospitals per 100,000 population, number of dentists per 100,000 population, unemployment rate, and percent of population with no health insurance coverage.
